# High Dietary Carbonyl Iron Reshapes the Gut Microbiome and Impairs Hepatic Insulin Sensitivity in a Time‐Dependent Manner

**DOI:** 10.1096/fj.202504722R

**Published:** 2026-02-24

**Authors:** Yupeng Li, Carine Fillebeen, Sinan Li, Jiarui Chen, Gary Sweeney, Kostas Pantopoulos

**Affiliations:** ^1^ Lady Davis Institute for Medical Research, Jewish General Hospital, and Department of Medicine McGill University Montreal Quebec Canada; ^2^ Guangdong Provincial Key Laboratory of Food, Nutrition and Health, and Department of Nutrition, School of Public Health Sun Yat‐Sen University Guangzhou People's Republic of China; ^3^ Department of Biology York University Toronto Ontario Canada

**Keywords:** α‐diversity, β‐diversity, insulin resistance, intestinal microbiome, iron metabolism

## Abstract

The gut microbiome is widely viewed as an important regulator of host metabolism and immunity. Loss of microbial diversity can lead to gut dysbiosis, which has been linked to cardiometabolic and inflammatory disorders. Iron is an important micronutrient for both host and microbes, but its excess is toxic. To investigate the impact of dietary iron on the intestinal microbiome and host metabolism, wild type mice on standard chow were switched at baseline to a high‐iron diet, containing 2% carbonyl iron for 3 weeks. Other groups of mice were switched to the high‐iron diet only during the final 3 or 7 days of the 3‐week period; control animals remained on standard chow. Fecal samples were collected at baseline (*t* = 0) and at the endpoint (*t* = 1) for microbiome analysis, while liver and skeletal muscle samples were analyzed for Akt phosphorylation as a marker of insulin sensitivity. Feeding with high carbonyl iron significantly altered the intestinal microbiome and increased overall alpha and beta diversity in a time‐dependent manner. Differential abundance and network analyses revealed extensive taxonomic and structural reorganization, with notable increases in *Akkermansiaceae*, *Rikenellaceae*, *Bilophila*, *Ruminiclostridium*, and *Lactobacillus*, and decreases in *Bifidobacteriaceae* and *Clostridiaceae_1*. Iron overload was accompanied by reduced Akt phosphorylation, evident in the liver but not skeletal muscles at the 3‐week endpoint. Together, these results demonstrate that feeding of mice with a high carbonyl iron diet reshapes gut microbial composition, increases diversity, and reorganizes microbial community networks. However, iron overload mitigates insulin responsiveness in the liver.

## Graphical abstract

High dietary carbonyl iron intake in mice reshapes the gut microbiome in a time‐dependent manner, increasing overall microbial diversity and reorganizing community networks. This is accompanied by selective enrichment of *Akkermansiaceae*, *Rikenellaceae*, *Bilophila*, *Lactobacillus*, and *Ruminiclostridium*, and depletion of *Bifidobacteriaceae* and *Clostridiaceae_1*. Despite increased microbiome diversity, iron overload selectively impairs hepatic insulin signaling, evidenced by reduced Akt phosphorylation, while skeletal muscle insulin responsiveness is preserved.

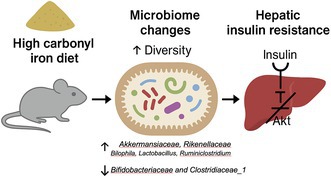

## 1 Introduction

The gastrointestinal tract is colonized by a large number of bacteria, fungi, parasites, and viruses [1, 2]. Collectively, they form the gut microbiome, a complex ecosystem that profoundly influences host physiology. Gut bacteria and the host coexist in finely tuned symbiotic relationships, which may be mutualistic, commensalistic, or parasitic. The host provides a stable niche and a continuous nutrient supply, while bacteria aid in food digestion and produce metabolites essential to the host that are protective against metabolic disorders [3]. The balance between host and microbiome is highly dynamic and depends on factors such as host genetics, epithelial barrier integrity, immune responses, diet composition, and environmental exposures. Disruption of this equilibrium leads to dysbiosis, characterized by reduced microbial diversity, loss of beneficial species, and expansion of pathogens. Dysbiosis has been linked to a broad spectrum of chronic diseases, including inflammatory bowel disease (IBD), diabetes, obesity, cardiovascular disease, metabolic dysfunction‐associated steatotic liver disease (MASLD), and neurological conditions [4, 5, 6, 7].

Recent studies emphasized the role of iron as a critical regulator of the gut microbiome [8, 9, 10, 11, 12]. Iron is an essential micronutrient for the host and almost all gut bacteria [13]. In mammals, large amounts of iron are required for the synthesis of heme, an integral part of the oxygen carrier hemoglobin in red blood cells. Non‐erythroid cells and bacteria utilize smaller amounts of iron as a cofactor in electron transfer and enzymatic reactions. To maintain adequate iron stores and compensate for non‐specific iron losses (e.g., via cell shedding or bleeding), the host relies on dietary iron intake [14]. Under physiological conditions, however, only a small fraction (~10%–15%) of ingested iron is absorbed, leaving the intestinal lumen enriched in iron available to bacteria.

While essential, excess iron is potentially toxic due to its redox reactivity and capacity to generate oxidative stress [15]. This is linked to intestinal inflammation and mucosal barrier disruption, which has important implications for iron supplementation to anemic IBD patients [16, 17]. Cells counter the challenges of iron toxicity by regulating iron uptake and activating antioxidant defense mechanisms. Because different bacterial species vary in their adaptive capacity, excess iron can act as a selective pressure, promoting the growth of pathogenic taxa (e.g., *Enterobacteriaceae, Clostridium*) and suppression of probiotic bacteria (e.g., *Bifidobacterium*, *Lactobacillus*) [17, 18, 19]. Furthermore, iron can alter bacterial short‐chain fatty acid production and bile acid metabolism, as well as host mucosal immunity [20, 21, 22]. Heme, which is more bioavailable compared to inorganic iron [14], appears to have a stronger impact on the gut microbiome [23]. It should also be noted that while luminal iron affects microbiome composition, bacterial metabolites can control host iron metabolism [24, 25].

The iron‐mediated crosstalk between the microbiome and the host may have significant pathophysiological ramifications [26]. There is evidence that iron status can affect liver steatosis through the gut microbiome in MASLD patients [27], while iron overload was reported to trigger adverse metabolic effects via the microbiota‐gut‐liver axis in a MASLD mouse model [28]. However, the mechanisms by which iron may specifically drive dysbiosis or metabolic dysfunction are not well understood. Herein, we utilized an established nutritional iron overload model, based on feeding mice a diet with a high (2%) carbonyl iron content, and investigated how excess iron intake alters gut microbiome composition and impacts host metabolism.

## 2 Materials and Methods

### 2.1 Experimental Animals and Sample Collection

Wild type C57BL/6J mice were housed in macrolone cages (up to 5 mice/cage, 12:12 h light–dark cycle: 7 a.m.–7 p.m.; 22°C ± 1°C, 60% ± 5% humidity). Male animals were fed until the age of 8 weeks (baseline) with a standard chow (2018 Teklad Global 18% protein, Envigo). Subsequently, groups of mice (*n* = 5–12 per group) were maintained on the standard chow (“Control”) or switched to a 2% carbonyl iron diet (CID; TD.09521, Envigo) for 3 weeks (“3‐Weeks CID”), or during the last 1 week (“1‐Week CID”) or 3 days (“3‐Days CID”) of the 3‐week period. At the endpoint, the animals were anesthetized by isoflurane and CO_2_ inhalation, sacrificed by cervical dislocation and exsanguinated by cardiac puncture. Where indicated, mice were injected via tail vein with 4 U/kg insulin 5 min before sacrifice. Fecal samples were collected at baseline (*t* = 0) and at the endpoint (*t* = 1). All fecal samples were snap‐frozen in liquid nitrogen immediately after collection and stored at −80°C until further analysis. Liver and skeletal muscle tissue samples were harvested, snap‐frozen, stored at −80°C and subsequently used for biochemical studies.

### 2.2 Analysis of Serum and Liver Iron Parameters

Serum was prepared and analyzed for iron, total iron binding capacity (TIBC), transferrin saturation, and ferritin by a Roche Hitachi 917 Chemistry Analyzer. Liver iron content was quantified by using a colorimetric ferrozine‐based assay [29].

### 2.3 Western Blotting

Liver and skeletal muscle tissue extracts were prepared in RIPA buffer containing 50 mM Tris, pH 8.0, 50 mM NaCl, 1% Triton X‐100, 0.5% sodium deoxycholate, 0.1% SDS, protease inhibitor tablet cOmplete Mini EDTA free (Sigma‐Aldrich) and a Halt phosphatase inhibitor Cocktail (Thermo Fisher Scientific). The suspensions were immediately homogenized with a tissue handheld homogenizer (Qiagen, Hilden, Germany). Cell debris was cleared by centrifugation, and the protein concentration was quantified with the DC‐Protein Assay (Bio‐Rad Laboratories, Hercules, CA). Lysates containing 40 μg protein were boiled in a loading buffer and resolved by SDS–PAGE on 9%–13% gels; subsequently, proteins were transferred onto nitrocellulose membranes (BioRad). The blots were blocked in either non‐fat milk or bovine serum albumin (BSA) diluted in tris‐buffered saline (TBS) containing 0.1% (v/v) Tween‐20 (TBS‐T) and probed overnight with antibodies against total Akt (1:1000 diluted, Cell Signaling #9272), p‐Akt Ser473 (1:1000 diluted, Cell Signaling #4060) or β‐actin (1:2000 diluted, Sigma). After the overnight primary antibody incubation, the membranes were washed 3× with TBS‐T and incubated with either 1:20 000 goat anti‐rabbit IgG or 1:5000 diluted peroxidase‐coupled goat anti‐mouse IgG (Jackson ImmunoResearch Laboratories). Immunoreactive bands were detected by chemiluminescence with a Western Lightning ECL Kit (Perkin Elmer, Waltham, MA, USA).

### 2.4 Glucose Tolerance Test

Mice subjected to dietary iron manipulations were fasted for 4 h. Subsequently, the animals were injected intraperitoneally with 2 g/kg glucose. Blood samples were collected from the tail vein at *t* = 0, 15, 30, 60 and 120 min post‐injection, and blood glucose levels were measured using the OneTouch Verio Flex blood glucose meter.

### 2.5 Processing of Microbiome Data

16S sequencing was carried out using Illumina MiSeq v2 at the McGill Genome Center, and Amplicon Sequence Variant (ASV) data were obtained. The relative abundances of microbiota were then calculated at the phylum, family, and genus levels. Moreover, taxonomic features with a detection rate lower than 10% were filtered out for further analysis.

### 2.6 Microbial Community Diversity Analysis

The Shannon diversity index, a pivotal metric for alpha diversity analysis, was calculated for each sample using the vegan package (v. 2.6.8). Paired comparisons between the baseline and endpoint for each diet group (“Control”, “3‐Days CID”, “1‐Week CID”, “3‐Weeks CID”) were conducted using the Wilcoxon signed‐rank test, a non‐parametric paired test, within a relevant package for statistical analysis (v. 0.6.0). To address potential future analyses and to ensure the robustness of the findings, the Benjamini–Hochberg false discovery rate (FDR) correction was applied across all experimental groups. Statistical significance was indicated by an FDR‐adjusted *p*‐value (*q*‐value) below 0.05.

Beta diversity, defined as a measure of the dissimilarity between microbial communities, was assessed using Bray–Curtis distances calculated with the vegan package (v. 2.6.8). This metric quantifies compositional differences based on the relative abundance of taxa. For each diet group, Bray–Curtis distances were computed between matched baseline and endpoint samples. To compare inter‐group differences in community structure changes, Wilcoxon rank‐sum tests with the Benjamini–Hochberg FDR correction for multiple comparisons were performed. Statistical significance was defined as *q* < 0.05 (FDR‐adjusted *p*‐value).

### 2.7 Differential Abundance Analysis

Differential abundance analysis at each taxonomic level was performed using MaAslin2 (v. 1.18.0) with a compound Poisson lognormal (CPLM) model. The model was constructed with Timepoint as the fixed effect, without data transformation or normalization. To address the compositional nature of microbiome data, a supplementary validation analysis was performed using ANCOM‐BC (v. 2.10.1). This model was constructed with Timepoint as the fixed effect, using the default bias‐corrected approach. Multiple testing correction was applied using the Benjamini–Hochberg method, and adjusted *p*‐values (*q*‐values) < 0.05 were considered statistically significant for differential features between baseline (*t* = 0) and endpoint (*t* = 1) within each experimental group.

### 2.8 Network Construction

Co‐abundant microbial networks for each dietary group at baseline and endpoint were constructed using the Family and Genus level taxonomic profiles. To ensure the biological relevance and statistical robustness of inferred interactions, a dual‐criteria threshold was applied for edge selection: (1) a moderate‐to‐strong correlation strength (Spearman |*ρ*| > 0.3), and (2) a nominal statistical significance (unadjusted *p*‐value < 0.05). This approach prioritizes associations with meaningful effect sizes while controlling for random noise. The construction and analysis of the co‐abundant networks were performed using the igraph package (v. 2.0.3), which facilitated the calculation of four topological parameters: node degree, betweenness centrality, closeness centrality, and local clustering coefficient. The bootstrap enhanced Kolmogorov–Smirnov test (10 000 iterations, *α* = 0.05) in the matching package (v. 4.10.15) was utilized to evaluate the temporal variation of topological properties. Additionally, to contextualize the robustness of network inference given the sample size (*n* = 6 per group), a supplementary bootstrap resampling analysis (1000 iterations) was performed using the “boot” package (v. 1.3.32). This confirmed the expected variability in network topology under such sampling constraints.

### 2.9 Exploration of Potential Biomarkers

NetMoss analysis was employed to identify potential biomarkers in microbial networks. A liberal significance threshold (adjusted *p* < 0.2) was used for initial screening to capture a broader set of taxa for subsequent integration. The results were integrated with those from the previous differential abundance analysis to identify key bacteria with significant differences between the “Control” and “3‐Weeks CID” groups. The abundance changes of these key bacteria between baseline and endpoint were visualized to understand their response to the dietary intervention.

### 2.10 Visualization

Visualization was mainly conducted with ggplot2 (v. 3.5.1) in R, and the co‐abundant networks were subjected to mapping in Cytoscape (v. 3.10.2).

## 3 Results

### 3.1 Exposure of Mice to a 2% Carbonyl Iron Diet

**FIGURE 1:**
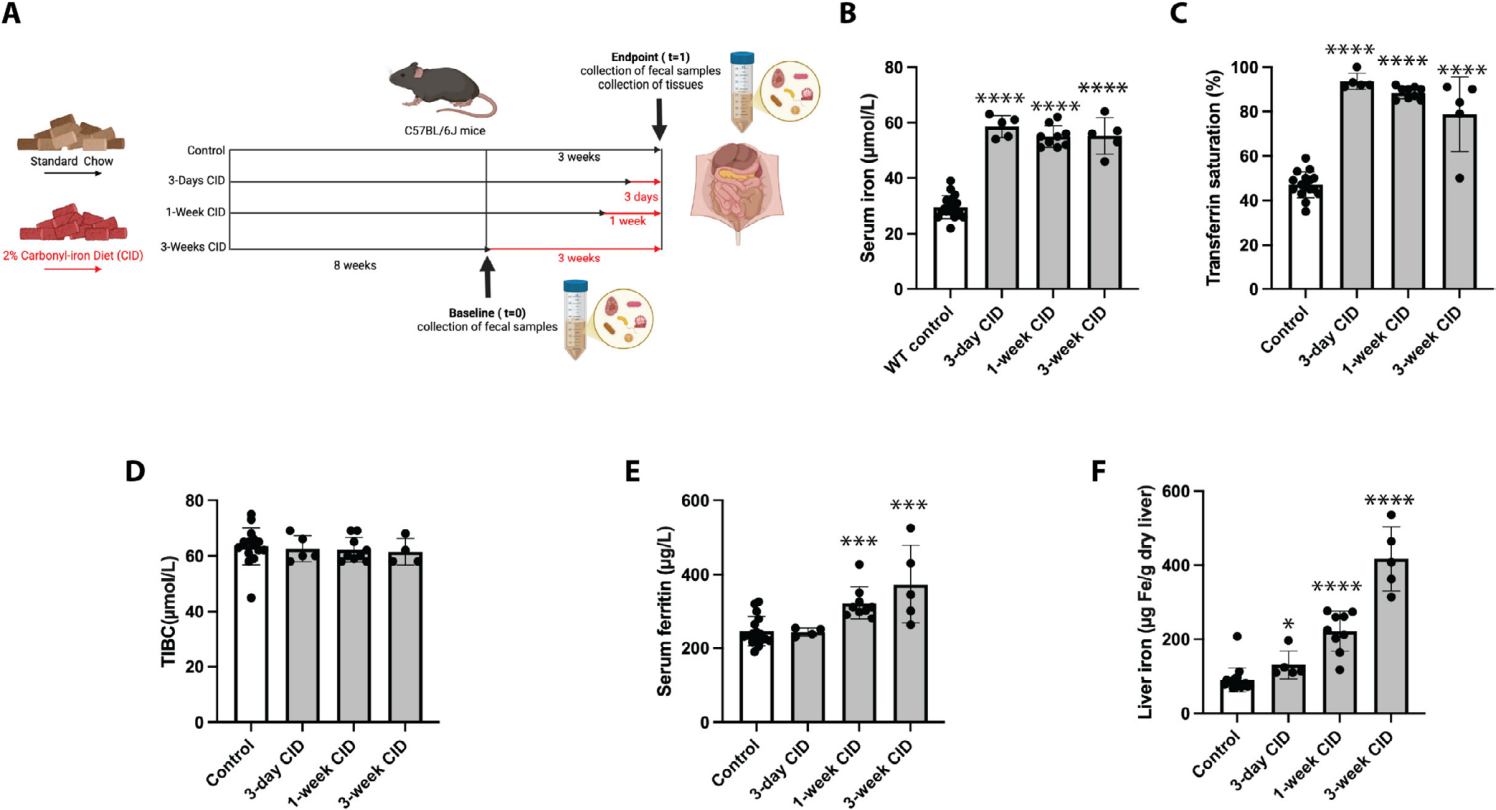
Intake of a 2% carbonyl iron diet causes systemic iron overload. Male C57BL/6J mice, fed until the age of 8 weeks (baseline) with a standard chow, were randomly divided in experimental groups (*n* = 5–12 per group). Mice in the “Control” group were maintained on the standard chow for another 3 weeks, while mice in the “3‐Weeks CID”, “1‐Week CID” and “3‐Days CID” groups were switched to a 2% carbonyl iron diet (CID) for 3 weeks, or during the last 1 week or 3 days of the 3‐week period. At the endpoint the mice were euthanized, and serum and tissues were collected for biochemical studies. (A) Schematic representation of experimental outline; created in BioRender. Li (2025), https://BioRender.com/4ibqywz. (B) Serum iron. (C) Transferrin saturation. (D) Total iron binding capacity (TIBC). (E) Serum ferritin. (F) Liver iron content. Quantitative data are presented as the mean ± SEM. Statistical differences (*p* < 0.05) were determined using one‐way ANOVA with the GraphPad Prism software (version 10.6.1). *p < 0.05; ***p < 0.001; ****p < 0.0001.

Male wild type mice were fed a 2% CID for 3 weeks or during the last 1 week or 3 days of the 3‐week period; control mice remained on standard chow (Figure 1A). As expected, CID intake led to a rapid increase in serum iron levels and transferrin saturation already within 3 days (Figure 1B,C) without changes in TIBC (Figure 1D). Furthermore, it triggered a gradual increase over time in serum ferritin levels and liver iron content (Figure 1E,F).

### 3.2 Basic Structure of Gut Microbial Communities and Iron‐Induced Changes

**FIGURE 2:**
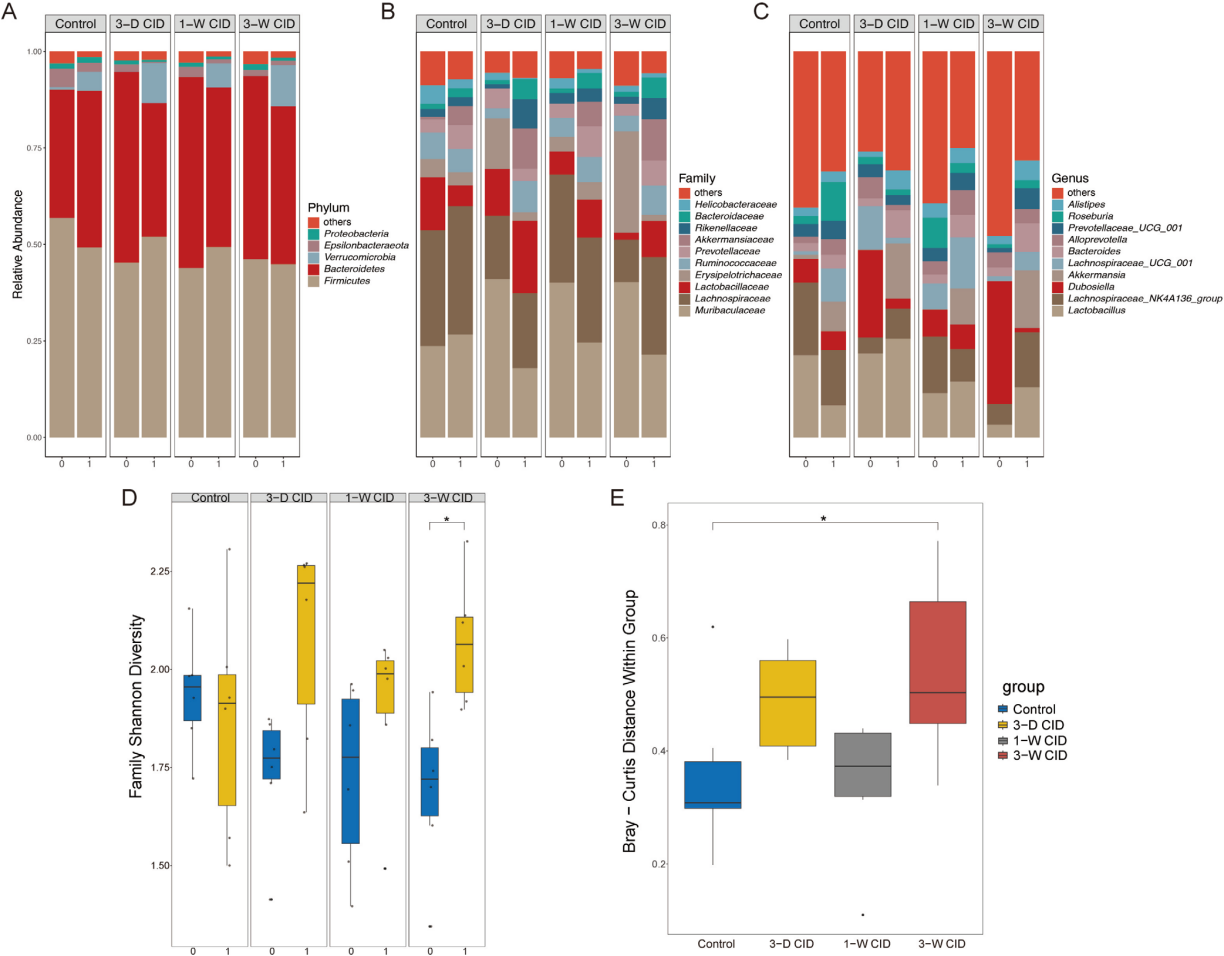
Taxonomic and diversity shifts in the gut microbiota of experimental mice following CID intake. Fecal samples from mice described in Figure 1 were collected at baseline (*t* = 0) and at the endpoint (*t* = 1). Experimental (A–C) Comparison of Microbial Composition at Phylum, Family and Genus Levels at baseline (*t* = 0) and at the endpoint (*t* = 1). (D) Box plot of Shannon alpha index analysis at the Family level. (E) Box plot of Bray–Curtis distances analysis between *t* = 0 and *t* = 1 at the Family level. *q < 0.05.

Fecal samples obtained at baseline and at the endpoint were used to assess the impact of CID feeding on the gut microbiome. At the phylum level and at baseline, this was dominated by *Firmicutes* and *Bacteroidetes*. Three weeks of CID feeding reduced the relative abundance of *Bacteroidetes* but did not significantly affect that of *Firmicutes*; this pattern was distinct from control mice (Figure 2A). At the family level, *Muribaculaceae* and *Lachnospiraceae* were the most abundant; the former was negatively affected by iron already on Day 3 of CID feeding (Figure 2B). Likewise, at the genus level, *Dubosiella* decreased in response to CID feeding. In parallel, *Akkermansia* and *Lactobacillus* showed an increase across all CID groups (Figure 2C). Collectively, these findings indicate that short‐term, as well as prolonged CID feeding reshapes the composition of the gut microbiome.

### 3.3 Impact of CID on Gut Microbiome Diversity

Short‐term (3 days) or prolonged (1–3 weeks) CID feeding were both associated with a significant increase in the Shannon index (Figure 2D), reflecting higher alpha diversity. Consistent with this, Bray–Curtis distance analysis revealed a significant iron‐induced increase in beta diversity across groups (Figure 2E). Thus, CID feeding appears to increase the gut microbiome diversity within the 3‐week experimental time frame.

### 3.4 Bacteria With Significantly Altered Relative Abundance

**FIGURE 3:**
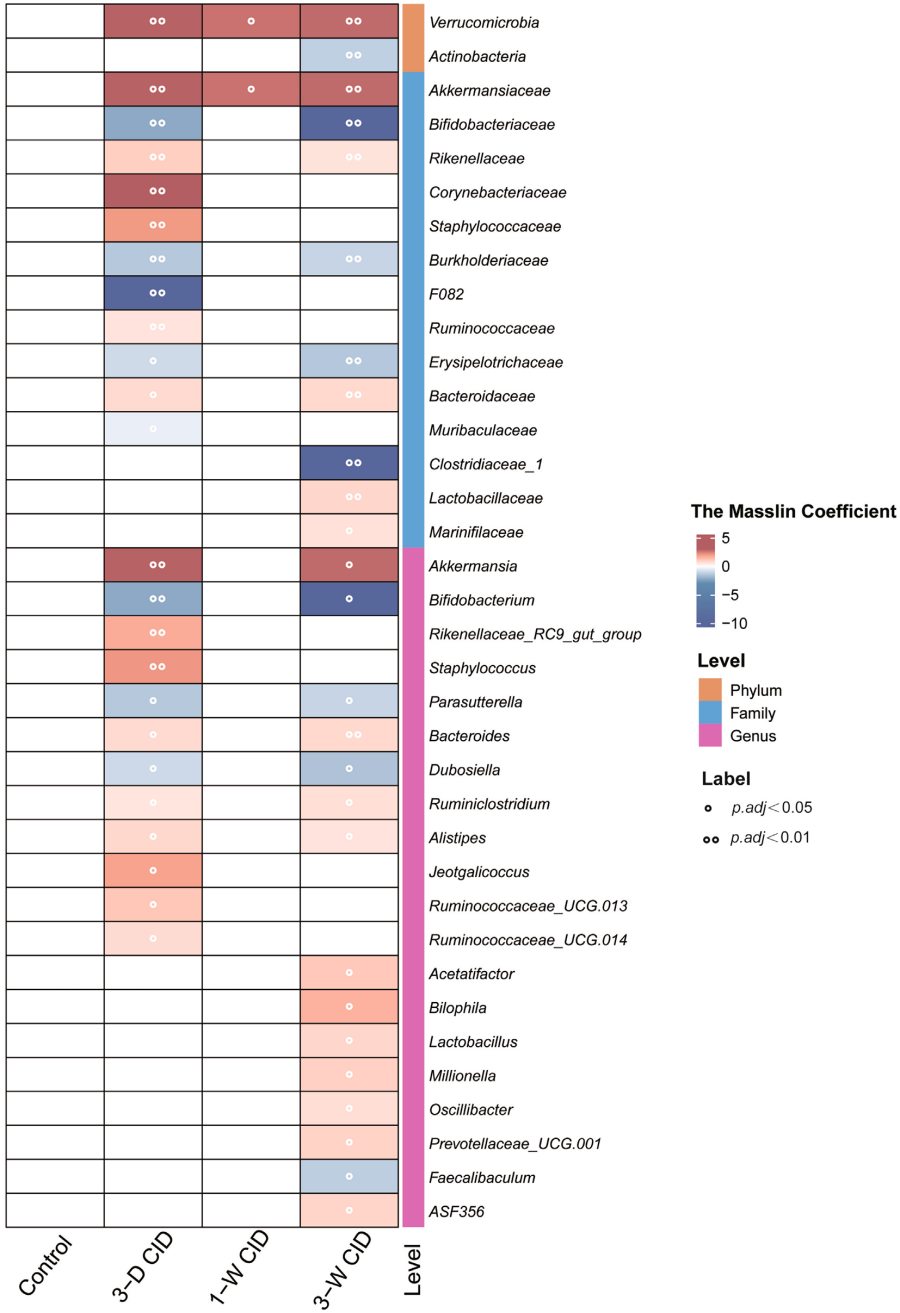
Differential abundance heatmap of gut microbiota taxa with significant shifts in relative abundance following CID intake. Experimental conditions are described in Figures 1 and 2.

To identify taxa with significant changes in relative abundance, we performed MaAslin2 analysis and visualized the results in a heatmap (Figure 3). At the phylum level, two taxa were altered: *Verrucomicrobia* increased, while *Actinobacteria* decreased. At the family level, 14 taxa were affected, with 8 showing increased abundance and 6 showing decreased abundance. At the genus level, 20 taxa were identified, of which 16 increased and 4 decreased in abundance.

We conducted a supplementary analysis using ANCOM‐BC to further examine the microbial changes. The results were largely consistent with those from the primary MaAslin2 analysis: *Verrucomicrobia* remained the most significantly altered phylum, 6 of the 14 differentially abundant families were confirmed, and seven of the 20 genera were corroborated (Figure S1). This consistency between analytical approaches validates the robustness of the observed microbial alterations associated with CID feeding.

### 3.5 CID Feeding Alters the Network Structure of Intestinal Flora

**FIGURE 4:**
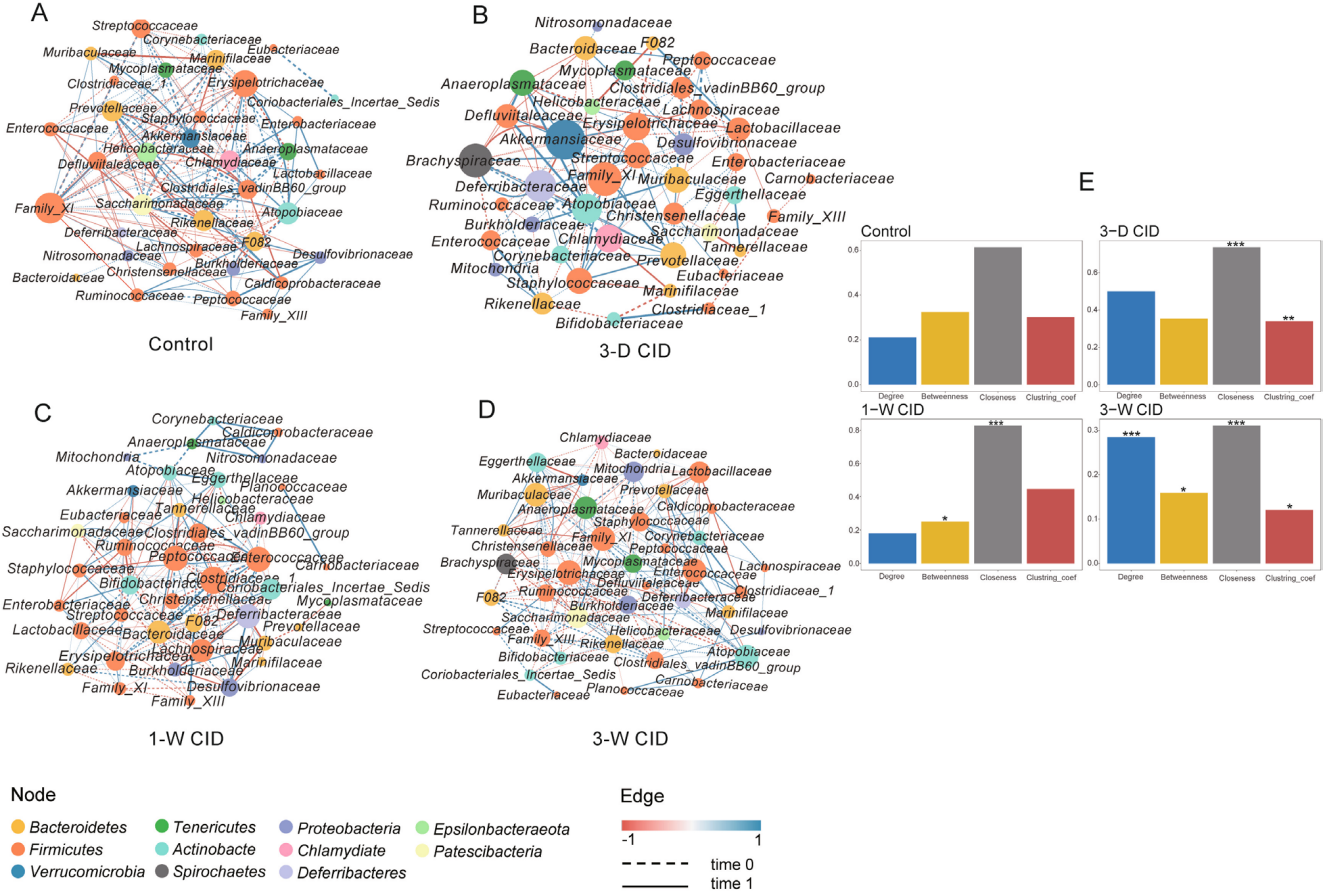
Network correlation dynamics and topological alterations in gut microbiota following CID intake at the Family level. Experimental conditions are described in Figures 1 and 2. (A–D) Network of Family level. The thickness of the edge reflects the magnitude of the correlation. The size of the node represents the magnitude of the Node Degree. (E) Bar chart of Network property indices. *p < 0.05; **p < 0.01; ***p < 0.001.

We next analyzed microbial community network structures at the family and genus levels, constructing networks at baseline and endpoint using a dual‐criteria threshold (|*ρ*| > 0.3 and *p* < 0.05) to identify robust associations. To assess the temporal stability of microbial interactions, we identified associations that met these criteria at both time points with a consistent direction (positive or negative). At the family level, such robust and persistent interactions constituted 4.8% of all possible node pairs in the control group. This proportion decreased to 3.9% in the “1‐Week CID” group and further to 2.4% in the “3‐Weeks CID” group (Figure 4A–D), suggesting a reduction in stable microbial associations with prolonged CID feeding. Analysis of network properties revealed no statistically significant differences between baseline and endpoint in controls. However, as the duration of CID feeding increased, the number of indices showing considerable differences also increased, with all indices (node degree, betweenness centrality, closeness centrality, and local clustering coefficient) reaching statistical significance in the “3‐Weeks CID” group (Figure 4E).

**FIGURE 5:**
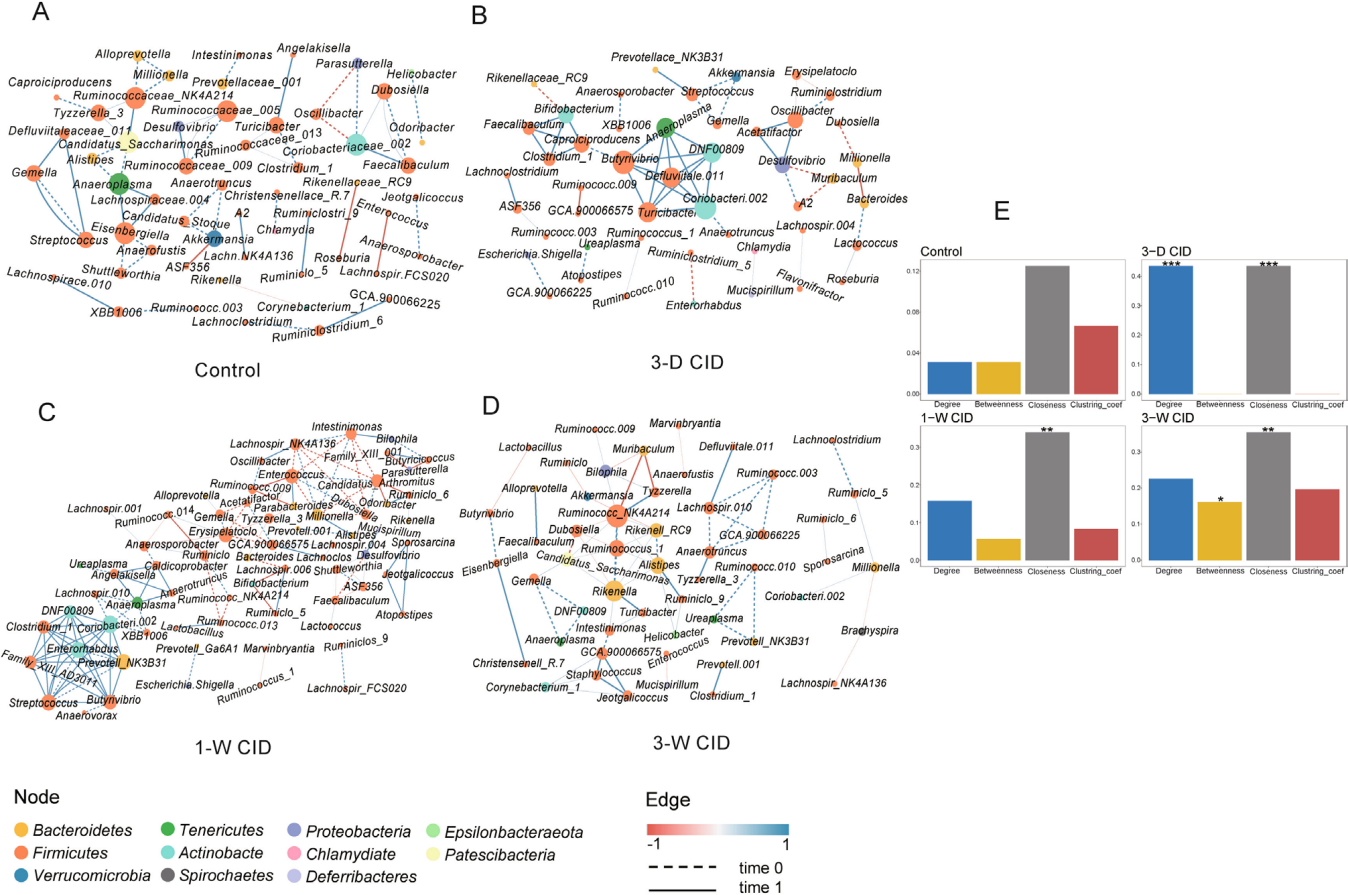
Network correlation dynamics and topological alterations in gut microbiota following CID intake at the Genus level. Experimental conditions are described in Figures 1 and 2. (A–D) Network of Genus level. The thickness of the edge reflects the magnitude of the correlation. The size of the node represents the magnitude of the Node Degree. (E) Bar chart of Network property indices. *p < 0.05; **p < 0.01; ***p < 0.001.

At the genus level, the proportion of robust and persistent interactions decreased from 5.7% in controls to 2.4% in the “1‐Week CID” group, with no such interactions meeting the criteria in the “3‐Weeks CID” group (Figure 5A–D). Importantly, the network property results at the genus level (Figure 5E) were consistent with those observed at the family level (Figure 4E). Together, these findings indicate that longer durations of CID feeding trigger more substantial reorganization of microbial community networks.

### 3.6 Exploration of Potential Biomarkers for Iron‐Induced Alterations in the Intestinal Microbiome

**FIGURE 6:**
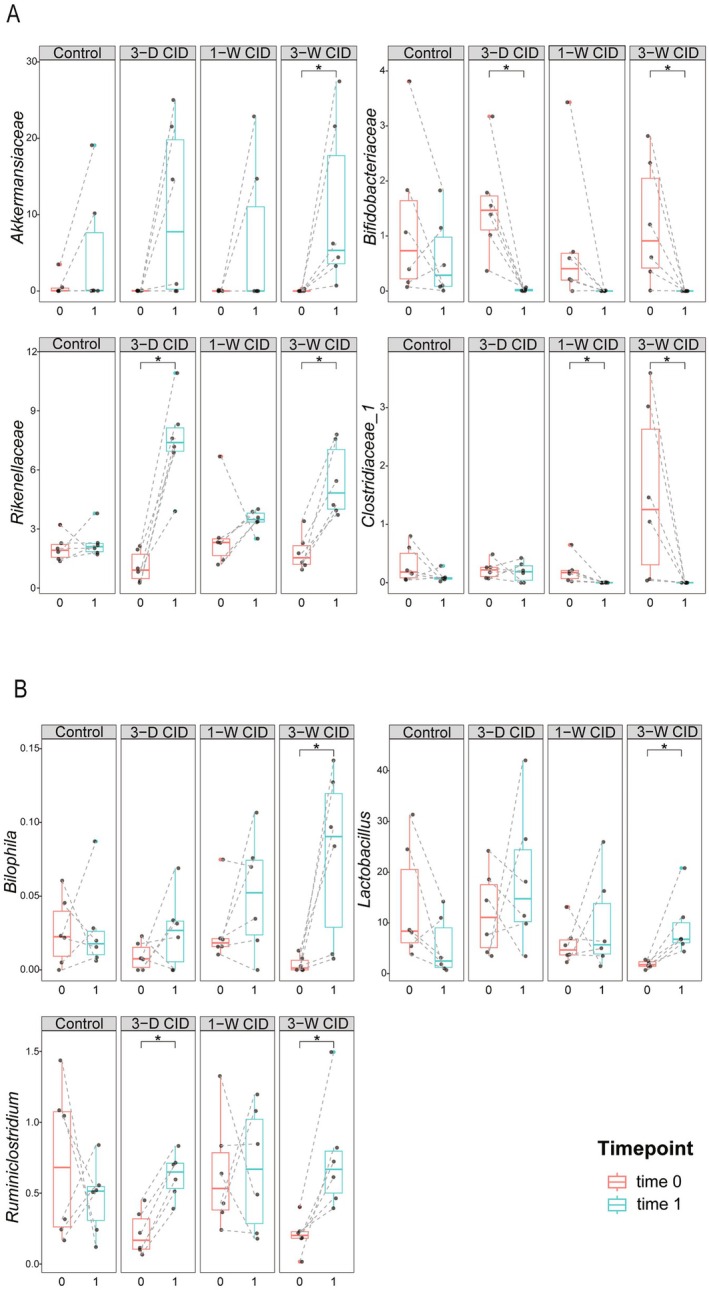
Longitudinal analysis of CID‐induced shifts in relative abundance of key bacterial taxa at Family and Genus levels. Experimental conditions are described in Figures 1 and 2. (A) Four key bacteria at the Family level (*Akkermansiaceae*, *Bifidobacteriaceae*, *Rikenellaceae*, and *Clostridiaceae_1*). (B) Three key bacteria at the Genus level (*Bilophila*, *Lactobacillus*, and *Ruminiclostridium*).

NetMoss analysis was applied as an exploratory approach to identify potential candidate biomarkers (adjusted *p* < 0.2, Tables S1 and S2). At the family level, taxa with increased NetMoss values in the “3‐weeks CID” group, relative to controls, were identified. Integrating these results with the previous differential abundance analysis, four key families were selected for further investigation (Figure 6A). At the genus level, three key taxa were identified using the same approach (Figure 6B). Analysis of relative abundance changes between baseline and endpoint revealed that 3‐week CID feeding increased the abundances of *Akkermansiaceae* and *Rikenellaceae* at the family level, and *Bilophila*, *Lactobacillus*, and *Ruminiclostridium* at the genus level. In contrast, it reduced the abundances of *Bifidobacteriaceae* and *Clostridiaceae_1* at the family level (Figure 6A,B).

### 3.7 Mice Fed the CID Eventually Develop Signs of Insulin Resistance in the Liver but Not in Skeletal Muscles

**FIGURE 7:**
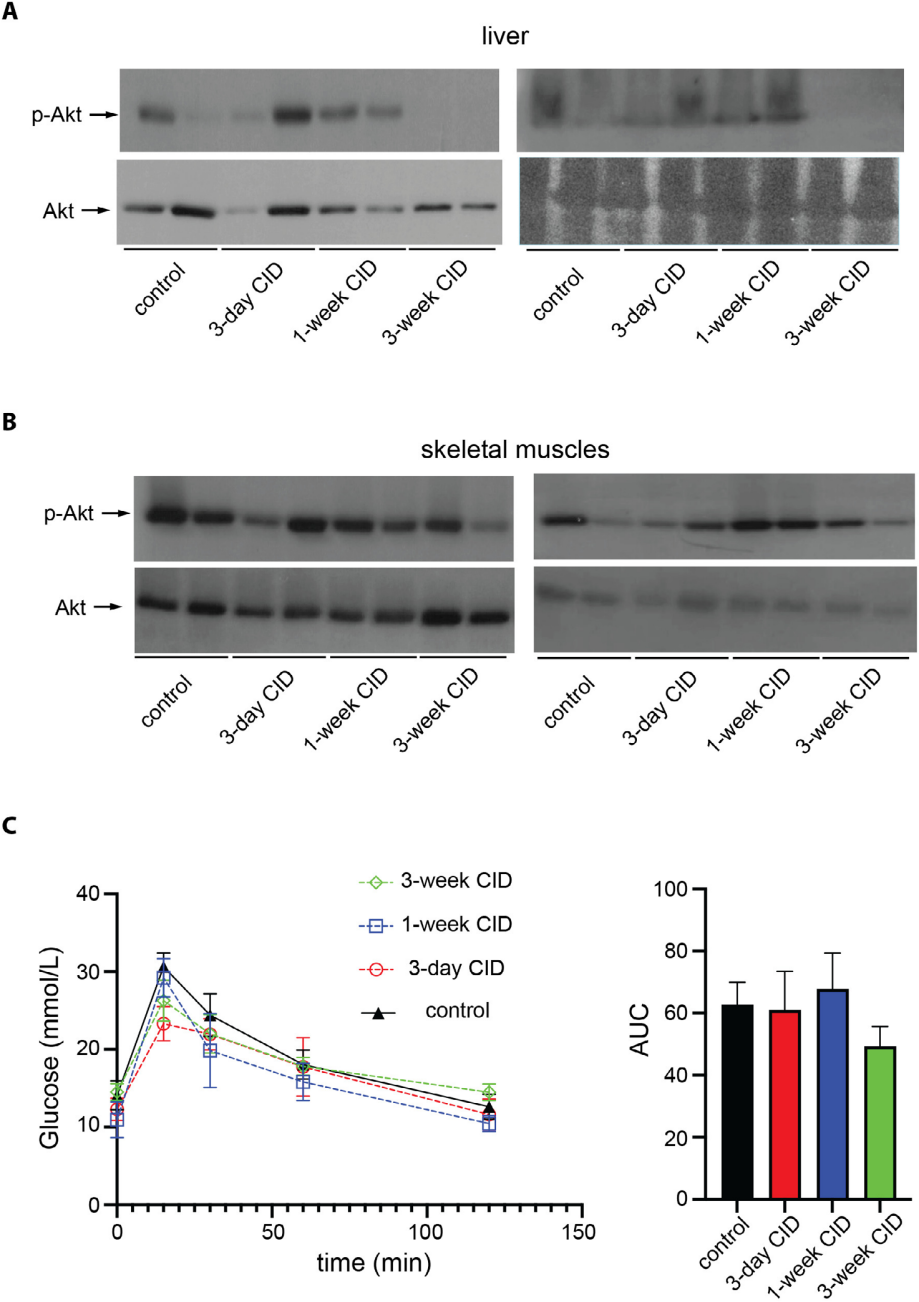
CID intake induces time‐dependent insulin resistance in the liver but not in skeletal muscles. Mice were subjected to dietary iron manipulations as described in Figure 1. In (A, B), the animals were injected via tail vein with 4 U/kg insulin 5 min before sacrifice. Liver and skeletal muscle extracts were prepared and analyzed by Western blotting for expression of p‐Akt and Akt. (A) p‐Akt and Akt expression in the liver. (B) p‐Akt and Akt expression in skeletal muscles. The blots represent data from 4 biological replicates in each experimental group. (C) Mice were subjected to glucose tolerance test following fasting for 4 h; area under curve (AUC) is shown on the right.

We examined the effects of CID intake on insulin sensitivity in the liver and skeletal muscles, two metabolically important tissues. Insulin responsiveness was assessed by monitoring Akt phosphorylation at S473 following insulin injection. Hepatic phospho‐Akt levels were unchanged after 3 days or 1 week CID feeding but were markedly reduced after 3 weeks (Figure 7A). In contrast, Akt phosphorylation in skeletal muscles was not significantly altered by CID at any time point (Figure 7B). Along these lines, glucose tolerance tests revealed no significant differences in glucose disposal following CID intake (Figure 7C). Together, these results indicate that CID induces a time‐dependent impairment of hepatic insulin signaling, whereas skeletal muscles remain insulin‐responsive and systemic glucose clearance is preserved during the 3‐week experimental period.

## 4 Discussion

We show that short‐term and prolonged dietary intake of 2% carbonyl iron induces significant shifts in the intestinal microbiome of mice. Unexpectedly, overall α‐ and β‐diversity increased, despite reductions in *Bifidobacteriaceae* and *Clostridiaceae_1*. Probiotic supplementation with *Bifidobacterium* is considered to offer therapeutic benefits against metabolic disorders; however, a recent systematic review and meta‐analysis suggested that this only applies to animal studies and not to clinical trials [30]. Even though *Clostridium* species possess probiotic characteristics and are known regulators of intestinal homeostasis [31], our data suggest that CID intake does not induce dysbiosis in mice. This is also supported by the CID‐induced increased abundance of *Lactobacillus*, a genus of bacteria that appears to improve metabolic health [32].

Excess luminal iron is generally thought to exert detrimental effects on the microbiome by favoring the expansion of pathogenic species. For example, iron fortification in Kenyan infants enhanced the growth of enteropathogens and was associated with intestinal inflammation [33], while in patients with inflammatory bowel disease, oral but not intravenous iron therapy reduced gut bacterial diversity [34].

In mice, dietary ferrous sulfate (up to 500 mg/kg) did not markedly affect overall α‐diversity, although it reshaped the abundance of specific taxa [35]. Notably, different forms of dietary iron, even when provided at identical doses (50 mg/kg) and yielding similar luminal iron concentrations, can elicit distinct microbiome responses. Unlike ferrous sulfate, ferrous bisglycinate and ferric EDTA reduced α‐diversity and differentially modulated the severity of dextran sodium sulfate (DSS)‐induced colitis [35]. Among various iron sources, heme (50 mg/kg) appears to provoke the most pronounced dysbiosis, producing diversity shifts comparable to those seen in DSS‐treated mice and exacerbating DSS‐induced colitis [36], whereas ferrous bisglycinate was protective [35].

Our findings align with the concept that different iron formulations distinctly influence gut microbial communities. Although ferrous salts and ferric polysaccharide complexes are widely used in iron‐replacement therapies, there is no clear evidence that the redox state of iron (ferrous vs. ferric) dictates the microbiome effects. Instead, the chemical ligands associated with each formulation likely contribute to their divergent impacts [35, 36, 37, 38].

Carbonyl iron, a form of elemental iron produced from vaporized iron pentacarbonyl complexes, is used at low doses as a food additive and, less commonly, as an iron supplement [39]. Whether (and how) these lower exposures alter the gut microbiome remains unknown. Thus, it is intriguing that the much higher levels of carbonyl iron provided in the CID increase rather than decrease bacterial diversity in our mouse model. Considering the known selective toxicity of iron to certain bacterial populations, this finding is also counterintuitive and underlines the complexity in the iron‐microbiome crosstalk.

However, the increased bacterial diversity following CID intake can be rationalized within an ecological framework. Unlike soluble salts, which can cause abrupt and high local iron concentrations, the particulate and insoluble nature of elemental carbonyl iron likely results in a slow and sustained release of iron ions within the gut lumen. This may create moderate, selective pressure that suppresses certain iron‐sensitive taxa (potentially contributing to the observed reduction in *Bacteroidetes*), thereby releasing ecological niches and allowing for a more equitable distribution among remaining and potentially iron‐utilizing bacteria, ultimately increasing evenness and diversity. This concept is supported by a recent study in which iron‐saturated lactoferrin, another form of iron with controlled bioavailability, increased the alpha diversity of the gut microbiota, whereas the iron‐free form did not [40]. Thus, the CID‐induced shift likely reflects a community‐level adaptation to an altered yet not broadly toxic iron environment.

We also demonstrate that CID intake correlates with signs of insulin resistance in the liver. This is likely a direct effect of hepatic iron overload [41] rather than a consequence of increased gut bacterial diversity, which is rather known to promote metabolic homeostasis along the gut‐liver axis [1, 6]. Severe systemic iron loading, such as that induced by parenteral iron dextran, can also trigger insulin resistance in skeletal muscles through inhibition of late stage autophagic flux [42]. This response was not observed in our study, presumably due to the relatively modest iron accumulation in muscles following CID intake. Consistent with the preservation of insulin responsiveness in skeletal muscles, we found no significant CID‐related differences in blood glucose clearance. However, our data cannot exclude the possibility that CID may cause insulin resistance in other peripheral tissues. In another study, CID feeding induced systemic insulin resistance in *Citrobacter*‐infected mice, where elevated luminal glucose diminished bacterial virulence and protected the animals against a lethal enteric infection [43].

While our study establishes a clear association between CID feeding, specific taxonomic shifts, and host metabolic phenotypes, the precise functional implications of these microbial changes warrant further investigation. Given that our inferences are based on 16S rRNA gene sequencing—which robustly profiles taxonomy but has inherent limitations in predicting function—and considering the exploratory sample size (*n* = 6 per group) common in such mechanistic mouse studies, we interpret our findings as generating key hypotheses. For instance, the mechanistic links between CID‐altered taxa (e.g., increased *Akkermansia*, decreased *Bifidobacterium*) and hepatic insulin resistance require validation. Future studies employing larger cohorts, metagenomics, metabolomics, and gnotobiotic models are essential to confirm these functional connections, identify specific microbial metabolites, and delineate their causal roles within the gut‐liver axis during iron supplementation.

In conclusion, our data demonstrate that, in a mouse model, high dietary carbonyl iron intake is associated with unexpectedly increased microbiome diversity and with hepatic insulin resistance. Although causality remains to be determined, these findings reveal formulation‐specific effects of dietary iron on both microbial and metabolic outcomes and emphasize the need to better understand how distinct iron sources shape host–microbiome interactions and metabolic homeostasis.

## Author Contributions

Y.L.: investigation, formal analysis, methodology; C.F.: methodology, project administration; S.L.: formal analysis, methodology; J.C.: formal analysis, methodology, writing manuscript; G.S.: conceptualization, methodology, resources; K.P.: conceptualization, supervision, funding acquisition, writing manuscript.

## Funding

This work was supported by the Canadian Institutes of Health Research (CIHR) (PJT‐186193).

## Conflicts of Interest

The authors declare no conflicts of interest.

## Supporting information


**Figure S1:** ANCOM‐BC supplementary analysis of microbiome composition in response to CID intake. Experimental conditions are described in Figure 1 and Figure 2.

**Table S1:** NetMoss results at the Family level.

**Table S2:** NetMoss results at the Genus level.

## Data Availability

The metagenomics data are available at the NCBI database (BioProject ID: PRJNA1376141).
