# Supplementary material for: High Dietary Carbonyl Iron Reshapes the Gut Microbiome and Impairs Hepatic Insulin Sensitivity in a Time‐Dependent Manner
Source: FASEB J. 2026 Feb 24;40(4):e71626. doi: 10.1096/fj.202504722R (PMC12930339; doi:10.1096/fj.202504722R)
Supplement: Supplementary file 1 — Figure S1: ANCOM‐BC supplementary analysis of microbiome composition in response to CID intake. Experimental conditions are described in Figure 1 and Figure 2. [file FSB2-40-e71626-s003.pdf]

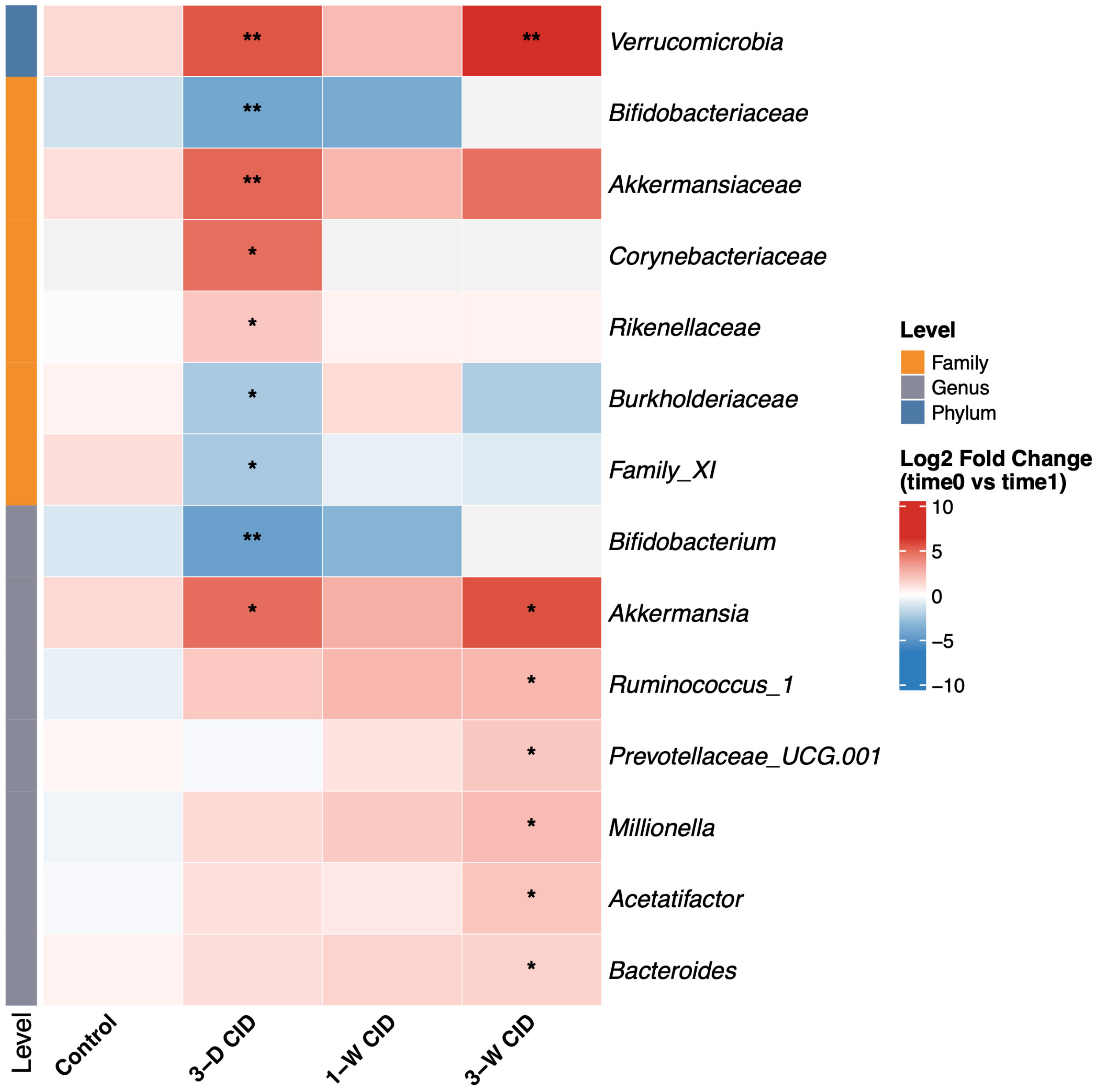

Fig. S1. ANCOM-BC supplementary analysis of microbiome composition in response to CID intake. Experimental conditions are described in Fig. 1 and Fig. 2.
