# Supplementary material for: High Dietary Carbonyl Iron Reshapes the Gut Microbiome and Impairs Hepatic Insulin Sensitivity in a Time‐Dependent Manner
Source: FASEB J. 2026 Feb 24;40(4):e71626. doi: 10.1096/fj.202504722R (PMC12930339; doi:10.1096/fj.202504722R)
Supplement: Supplementary file 2 — Table S1: NetMoss results at the Family level. [file FSB2-40-e71626-s002.pdf]

Table S1. NetMoss results at the Family level

Node (bacteria)	Control	3-D CID	1-W CID	3-W CID
Akkermansiaceae	0.13326011	0	0.68771707	0.36469803
Anaeroplasmataceae	0.11823503	0	0.86073238	0.5165774
Atopobiaceae	0.96526298	0	0.99367898	0.92065266
Bifidobacteriaceae	0.39995666	0	0	0.41220058
Burkholderiaceae	0.40976273	0	0.63730007	0.17475855
Caldicoprobacteraceae	0.99552764	0	0.86073238	1
Christensenellaceae	0.24242373	0	0	0.18307603
Clostridiaceae_1	0.15588046	0	0.92689963	0.81233391
Clostridiales_vadinBB60_group	0.12772626	0	0	0.45255385
Corynebacteriaceae	0.11609067	1	0.821364	0.75482055
Eggerthellaceae	0.00337425	0	0.99367898	0.19513707
Enterobacteriaceae	1	1	0	0.04885275
Enterococcaceae	0.09026927	0	1	0
Erysipelotrichaceae	1	0	0.38295644	0.75147561
Eubacteriaceae	1	0	0.79445193	0
Family_XI	0.10785287	0	0.48041038	0.73379323
Helicobacteraceae	0.15433244	0	0.35433205	0.46561048
Marinifilaceae	0.21219407	0	0.44562919	0
Mitochondria	0.15373392	0	0	0.60315164
Muribaculaceae	0.08342447	0	0.64712867	0.16942458
Mycoplasmataceae	0.12933695	0	0.27178095	0.89292073
Nitrosomonadaceae	0.116338	0	0.821364	0
Planococcaceae	0.05760163	0	0	0.42121404
Prevotellaceae	0.39000781	0	0.53057748	0
Rikenellaceae	0.06270166	0	0.7479331	0.22799678
Saccharimonadaceae	0.50565685	0	0.71174164	0.73640998
Staphylococcaceae	0.09097629	0	1	0.7941701
Streptococcaceae	0.24349342	0	0.77132879	0.23434492
Tannerellaceae	0.09911433	0	1	0.06482905
Defluviitaleaceae	0	0.2981812	0	0
Bacteroidaceae	0	0	0.92689963	0
Coriobacteriales_Incertae_Sedis	0	0	0.77990048	0.48536647
Family_XIII	0	0	0.54627253	0.37648169
Lactobacillaceae	0	0	0.30369959	0
Ruminococcaceae	0	0	0.74483572	0.30728332
Brachyspiraceae	0	0	0	0.03338835
Carnobacteriaceae	0	0	0	0.68754887
Deferribacteraceae	0	0	0	0.04023787
F082	0	0	0	1
