# Supplementary material for: High Dietary Carbonyl Iron Reshapes the Gut Microbiome and Impairs Hepatic Insulin Sensitivity in a Time‐Dependent Manner
Source: FASEB J. 2026 Feb 24;40(4):e71626. doi: 10.1096/fj.202504722R (PMC12930339; doi:10.1096/fj.202504722R)
Supplement: Supplementary file 3 — Table S2: NetMoss results at the Genus level. [file FSB2-40-e71626-s001.pdf]

Table S2. NetMoss results at the Genus level

Node (bacteria)	Control	3-D CID	1-W CID	3-W CID
A2	0.17726883	0.2301645	0	0.95452361
ASF356	0.48939925	0	0.40251253	0.41098791
Acetatifactor	0.46531074	0	0	0
Akkermansia	0.96536083	0	0	0.19681789
Alistipes	0.79051624	0.60026298	0.63476198	0
Alloprevotella	0.79514957	0	0	0
Anaerofustis	0.61425531	0.22519413	0	0
Anaeroplasma	0.84415104	0	0.46920541	0.63618528
Anaerosporobacter	0.65800039	0	0	0.88772903
Anaerotruncus	1	1	0.2366945	0.95513125
Anaerovorax	0.35843385	0	0	0
Angelakisella	0.7247298	0.42632015	0	0
Atopostipes	0.51564901	0.35156887	0	0.21825097
Bacteroides	0.23440705	0	0.90271953	0
Bilophila	0.30564669	0	0	0.82707084
Butyricicoccus	0.46246545	0	0	0
Caldicoprobacter	0.57491506	0	0.54880887	0.8232293
Candidatus_Arthromitus	0.72375414	0.39492515	0.50758408	0
Candidatus_Saccharimonas	0.97239374	0.09918361	0	0.8014133
Candidatus_Stoquefichus	1	0	0.26269097	0
Caproiciproducens	0.80936997	0	0.69452036	0
Chlamydia	0.90548275	0	0.28240309	0.72203972
Christensenellaceae_R.7_group	0.65599516	0	0	1
Clostridium_sensu_stricto_1	0.63791531	0	1	0.96129717
Coriobacteriaceae_UCG.002	0.92008517	0	0.86987454	0
Corynebacterium_1	0.9153485	0	0	1
DNF00809	0.46999505	0	0.69558038	0.93227525
Desulfovibrio	0.70489618	0.3645861	0	0
Dubosiella	0.97848047	0	0.34808617	0.8307396
Eisenbergiella	0.68892033	0	0.75778311	0.77948078
Enterococcus	0.77302758	0	1	0
Enterorhabdus	0.43797824	0	0	0.87852589
Erysipelatoclostridium	0.86640052	0	0.82303313	0
Escherichia.Shigella	0.83191965	1	0.10373842	0
Faecalibaculum	1	0	0.56135274	0.31323968
Family_XIII_AD3011_group	0.29010849	0	0.74395124	0.56950237
Family_XIII_UCG.001	0.17757193	0	0	0
Flavonifractor	0.56087979	0	0	0.16718311
GCA.900066225	0.95925381	0.60607752	0	0.72413433
Gemella	1	0	0.34734146	0.76535313
Helicobacter	0.69650816	0	0.32297156	0.82731728
Intestinimonas	0.88347271	0	0	0
Jeotgalicoccus	0.65800039	0	0	0.73192283
Lachnospiraceae_FCS020_group	0.73124518	0.32556399	0.36458546	0.4817551

Continued Table 2.

Node (bacteria)	Control	3-D CID	1-W CID	3-W CID
Lachnospiraceae_NK4A136_group	0.60966545	0.18676012	0	0
Lachnospiraceae_UCG.001	0.36568868	0	0	0.50096404
Lachnospiraceae_UCG.004	0.20333406	0	0	0
Lactobacillus	0.36743768	0	0.2701109	0.72448591
Lactococcus	1	0	0.67190461	0.2744008
Marvinbryantia	1	0	0.34367275	0.16057107
Millionella	0.63604613	0	0	0
Mucispirillum	0.18537414	0	1	0.76779814
Muribaculum	0.26730412	0	0	0.80659302
Odoribacter	0.75745975	0	0.22547074	0
Oscillibacter	0.60744436	0.50672999	0	0
Parabacteroides	0.16953627	0	0.86631083	0.74884132
Parasutterella	0.37993414	0	0.59267782	0.47931024
Prevotellaceae_Ga6A1_group	0.81200747	0	0	0
Prevotellaceae_NK3B31_group	0.45817922	0	0	0.97903376
Prevotellaceae_UCG.001	0.95009732	0	0	0.85106469
Rikenella	0.43274228	0	0	0
Rikenellaceae_RC9_gut_group	0.13790027	0	0	1
Roseburia	0.56954986	0.98372058	0	0
Ruminiclostridium_5	0.68810899	1	0	0
Ruminiclostridium_6	0.58579731	0	0	0.64075668
Ruminiclostridium_9	0.92238599	0	0	0
Ruminococcaceae_NK4A214_group	1	0.12118488	0	0
Ruminococcaceae_UCG.009	0.73093066	0.71050065	0.88466222	0.51681682
Ruminococcaceae_UCG.010	0.74798913	0	0	0.59082357
Ruminococcaceae_UCG.013	0.38108314	0	0.35015985	0.61120071
Ruminococcaceae_UCG.014	0.39919334	0	0	0.48632042
Ruminococcus_1	0.45717153	0.08787852	0	0.57321783
Shuttleworthia	0.06659999	0	0.40622365	0.25754616
Sporosarcina	0.08497718	0	0	0.42179354
Staphylococcus	1	0	0.37252749	1
Streptococcus	1	0	0	0.55044857
Turicibacter	0.55975435	1	0	0.88955978
Tyzzerella	0.26396892	0	0	0.84613353
Tyzzerella_3	0.8653431	0	0	0
Ureaplasma	0.43023404	0	0	1
XBB1006	0.13887924	0	0	0.31024995
Defluviitaleaceae_UCG.011	0	1	0	0.21719079
Lachnospiraceae_UCG.010	0	0.3701369	0	0.61614007
Ruminiclostridium	0	0.72845798	1	0.70780614
Ruminococcaceae_UCG.003	0	0.77752844	0.79831073	0.87699559
Brachyspira	0	0	0	1
Butyrivibrio	0	0	0	0.73316815
Lachnospiraceae_UCG.006	0	0	0	0.481854
